# Mitochondrial dynamics in diabetic peripheral neuropathy: Pathogenesis, progression, and therapeutic approaches

**DOI:** 10.1097/MD.0000000000042748

**Published:** 2025-07-18

**Authors:** Honghai Yu, Cunqing Yang, Guoqiang Wang, Xiuge Wang

**Affiliations:** a Department of Traditional Chinese Medicine, Hunan University of Medicine General Hospital, Huaihua, Hunan Province, China; b Department of Dermatology, Guang’anmen Hospital, China Academy of Chinese Medical Sciences, Beijing, China; c Postdoctoral Research Station, Guang’anmen Hospital, China Academy of Chinese Medical Science, Beijing, China; d Department of Endocrinology, Affiliated Hospital of Changchun University of Chinese Medicine, Changchun, Jilin Province, China.

**Keywords:** diabetic peripheral neuropathy, mitochondrial dynamics, pathologic mechanisms, pathophysiological

## Abstract

Diabetic peripheral neuropathy (DPN) is a chronic complication resulting from late-stage peripheral nerve damage in diabetes. It is associated with pain and can lead to foot ulcers and even amputations. Currently, there are no reversible treatments for DPN. The pathophysiology of DPN is extremely complex and involves multiple mediating factors. Despite extensive research by scholars worldwide, the exact mechanisms underlying DPN remain incompletely understood. Recent evidence increasingly supports the notion that dysregulation of mitochondrial fission and fusion proteins, which regulate mitochondrial morphology and quantity in neurons under hyperglycemic conditions, may be a key pathological mechanism of DPN. In fact, processes such as metabolism, energy production, inflammation, reactive oxygen species generation, and apoptosis rely on the balance between fission and fusion. Pathological alterations in this balance can lead to bioenergetic dysfunction and mitochondrial-mediated cell death, thus contributing to the progression of DPN. Mitochondria regulate their number, quality, and function through mitochondrial dynamics (fission and fusion) to maintain homeostasis and cope with structural and functional impairments under high-glucose conditions. This article discusses the pathophysiological changes in DPN, the role of mitochondrial dynamics in its pathogenesis, and current targeted mitochondrial therapies, aiming to enhance the understanding of the mechanisms involved in DPN and to explore more effective treatment methods and intervention strategies.

## 1. Introduction

Diabetes mellitus (DM) represents a significant global public health challenge, contributing to disability and premature mortality. According to the International Diabetes Federation, the number of individuals affected by DM exceeds 460 million worldwide, with projections suggesting it may rise to 628 million by 2045.^[1]^ A hallmark of DM is the severe microvascular and macrovascular complications that impact multiple organs and systems.^[2,3]^ Among these, diabetic peripheral neuropathy (DPN) is one of the most common chronic complications in both type 1 diabetes (T1DM) and type 2 diabetes (T2DM), affecting over half of all patients with DM.^[4]^ DPN primarily impacts sensory and motor nerves, leading to symptoms such as sensory abnormalities and even loss of sensation. Reports indicate that up to 25% of patients with chronic DPN may develop diabetic foot ulcers, with 5% of these cases requiring extensive surgical amputations.^[5]^ In addition, 20% to 30% of patients with DPN experience neuropathic pain.^[6,7]^ DPN is also closely associated with significant sleep disturbances, anxiety, and depressive symptoms, severely affecting patients’ quality of life and imposing considerable economic burdens on healthcare systems.^[8–10]^ Current treatment strategies for DPN focus on alleviating clinical symptoms, controlling blood sugar levels, providing neuroprotective support, and improving microcirculation. While strict glycemic control can enhance neuropathy outcomes in patients with T1DM, it does not reverse nerve damage and has limited effectiveness in preventing DPN in patients with T2DM.^[11]^ The Diabetes Control and Complications/Epidemiology of Diabetes Interventions and Complications trials showed that while intensive glucose control reduced the incidence of clinical neuropathy during the Diabetes Control and Complications Trial phase, all participants developed DPN in the Epidemiology of Diabetes Interventions and Complications follow-up phase. The “metabolic memory” effect, where past hyperglycemia causes irreversible neurotoxic damage, may explain this, with a strong link between hyperglycemia and DPN progression persisting 13 to 14 years later.^[12]^ As of now, there are no definitive therapies for the permanent prevention or reversal of DPN.^[13]^ Therefore, a better understanding of the underlying mechanisms of DPN and the identification of effective therapeutic strategies to halt its progression in the early stages of DM-related nerve injury are critical issues in DPN clinical management.

**Figure 1.**
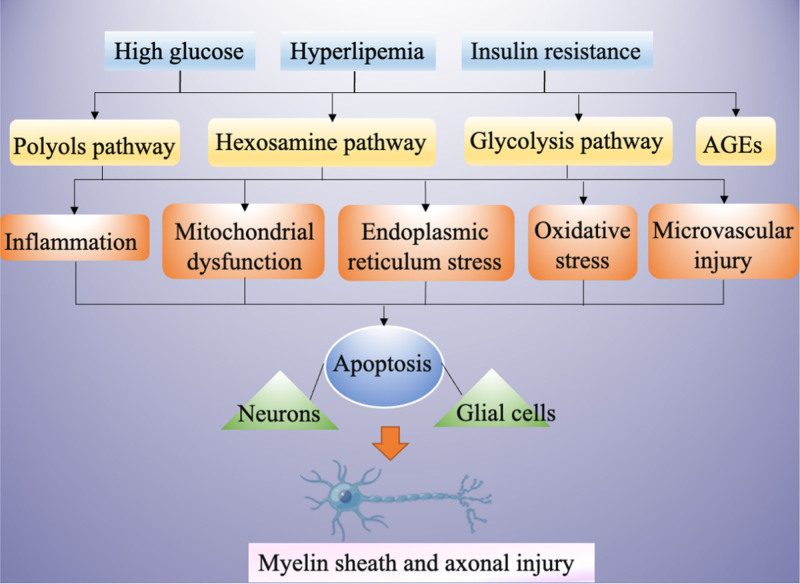
Classical mechanism of DPN. AGE = advanced glycation end-product, DPN = diabetic peripheral neuropathy.

Despite extensive research on DPN in recent years, its precise pathophysiological mechanisms remain unclear. However, several factors and pathways are known to be involved in the development of DPN. Hyperglycemia, hyperlipidemia, and insulin resistance have been reported as initiating factors for a cascade of pathophysiological changes leading to DPN. These conditions can trigger the activation of the polyol pathway, glycolytic pathway, hexosamine pathway, and advanced glycation end-products (AGEs) pathway.^[14]^ This cascade contributes to the release of proinflammatory cytokines, mitochondrial dysfunction, endoplasmic reticulum stress, accumulation of AGEs, and generation of reactive oxygen species (ROS). Furthermore, oxidative stress induced by AGEs can lead to microvascular damage.^[15]^ Concurrently, neuronal microvascular impairment obstructs blood supply to peripheral nerves, causing ischemic conditions that trigger abnormal apoptosis of neurons and glial cells.^[16]^ Ultimately, this results in demyelination of myelinated fibers, injury to unmyelinated fibers, and axonal atrophy, manifesting as impaired nerve conduction velocity and sensory dysfunction. This process significantly promotes the onset of diabetic neuropathy (Fig. 1).^[17–23]^

Recent research increasingly supports the notion that dysregulation of mitochondrial fission and fusion proteins, which control mitochondrial shape and quantity, is a key mechanism in the onset and progression of DPN under hyperglycemic conditions.^[24]^ The survival of neurons and their associated distal axons in the human peripheral nervous system is highly dependent on energy supply. The metabolic disturbances of glucose and lipid energy metabolism in adult sensory neurons, axons, and Schwann cells (SCs) caused by DM are central to the development of DPN. Mitochondria are at the intersection of critical cellular pathways, including energy substrate metabolism, ROS generation, and apoptosis.^[25]^ Mitochondrial dysfunction, particularly an imbalance in mitochondrial dynamics – encompassing both fusion and fission – inevitably leads to disruptions in energy homeostasis. Over time, this energy deficit can result in neurodegeneration of both neurons and axons.^[26]^ In fact, metabolism, energy production, calcium signaling, ROS generation, apoptosis, and aging all rely on the balance between mitochondrial fission and fusion. Pathological alterations in these processes can impair bioenergetic function and lead to mitochondrial-mediated cell death, thereby contributing to the progression of DPN.^[27,28]^ In both T1DM and T2DM animal models, an imbalance in the states of mitochondrial fusion and fission in peripheral nerves has been observed, along with impaired mitochondrial function and morphology, leading to insufficient ATP production and subsequent disruption of normal neural function. Furthermore, this imbalance can induce excessive ROS generation, triggering various metabolic disorders and oxidative stress, thereby accelerating the progression of DPN.^[29,30]^ In this review, we will focus on the various pathways through which mitochondrial dynamics influence the pathogenesis of DPN. In addition, we will discuss advancements in targeted therapies aimed at mitochondrial dynamics, with the goal of providing new therapeutic strategies for understanding the mechanisms of DPN and restoring mitochondrial homeostasis.

## 2. Pathophysiological change in DPN

DPN is a neurodegenerative disorder that primarily affects sensory and autonomic nerves, with motor nerves being involved to a lesser extent.^[31]^ DPN is often characterized as a length-dependent neuropathy, where the loss of distal epidermal axons in the calf occurs earlier than that in the proximal segments. Clinically, this manifests as “stocking and glove” symptoms, indicating that the longest sensory axons are the first to be affected.^[11]^ This phenomenon may be related to axonal degeneration associated with damage to myelin sheaths and SCs, leading to both primary and secondary demyelination.^[32,33]^ In myelinated and unmyelinated neurons, SCs become separated from the axons, disrupting the conduction of nerve impulses and signaling.^[34]^ Concurrently, a reduction in neurotrophic factors contributes to centripetal degeneration and distal axonal loss, with progression being dependent on the length of the fibers.^[34]^ Consequently, the longest nerve fibers, such as those in the sciatic and sural nerves, are at the greatest risk of injury.^[34,35]^ Progressive diabetic neuropathy reflects the atrophy and “death” of peripheral sensory axons, resulting in a range of sensory and motor functional abnormalities.

The pathophysiology of DPN is complex. Under chronic hyperglycemic conditions, 3 primary cell types are affected: neurons in both the peripheral and central nervous systems, SCs, and vascular cells. These cells have a limited capacity for glucose uptake and are therefore more susceptible to the effects of elevated glucose levels.^[36,37]^ Among these, dorsal root ganglion (DRG) neurons are the most significantly damaged cell type. Chronic hyperglycemia induces oxidative stress, metabolic abnormalities, and microvascular complications, which disrupt the normal structure and function of nerve cells through specific signaling pathways. This results in structural changes such as demyelination of neurons, Wallerian degeneration, and microvascular disease, ultimately leading to apoptosis of sensory DRG neurons. Consequently, there is damage and loss of both myelinated and unmyelinated fibers.^[38–42]^ SCs represent a crucial target in patients with DPN.^[43]^ Recent studies have revealed that SCs are involved in multiple pathways contributing to the development of DPN. In a diabetic state, hyperglycemia stimulates SCs to produce large amounts of ROS in their mitochondria, leading to mitochondrial DNA (mtDNA) damage and mutations, which ultimately result in SC death and axonal degeneration.^[44]^ Furthermore, SCs are closely related to inflammatory responses. Under hyperglycemic conditions, levels of proinflammatory cytokines such as tumor necrosis factor-alpha (TNF-α), interleukin-6 (IL-6), interleukin-1 beta (IL-1β), nuclear factor-kappa B (NF-κB), and toll-like receptors are significantly elevated within SCs. This may contribute to axonal degeneration and impair the interaction between axons and SCs.^[45]^ In addition, the secretion of various neurotrophic factors by SCs is reduced under hyperglycemic conditions, exacerbating neurodegeneration. Research has reported that in-vitro cultured mouse SCs show a downregulation of brain-derived neurotrophic factor expression levels under hyperglycemic conditions. This mechanism may be associated with the dephosphorylation of the protein kinase B (Akt)/mammalian target of rapamycin pathway.^[46]^ In addition, early in diabetes, hyperglycemia can induce abnormalities in blood flow and vascular permeability.^[47–50]^ Nukada et al^[50]^ observed swelling of endothelial cells in small endoneurial vessels from sural nerve biopsies, along with thickened basement membranes, leading to reduced vascular lumen size, they also noted a decrease in pericytes surrounding the endothelial cells.^[51]^ Consistent with this, another study confirmed the loss of pericytes and proliferation of endothelial cells in nerve tissue samples from patients with DPN.^[52]^ Damage to vascular cells in diabetes results in microvascular complications, which reduce intraneural blood flow, causing ischemia, hypoxia, and oxidative stress. These factors ultimately lead to loss of nerve tissue function and result in neurodegenerative necrosis.^[53]^ In summary, diabetic neuropathy may arise from both the direct effects of hyperglycemia on damaged cells^[54,55]^ and the indirect impacts on cellular function, thus promoting the development of DPN.^[47,56]^

## 3. Pathological mechanisms related to mitochondrial dynamics

**Figure 2.**
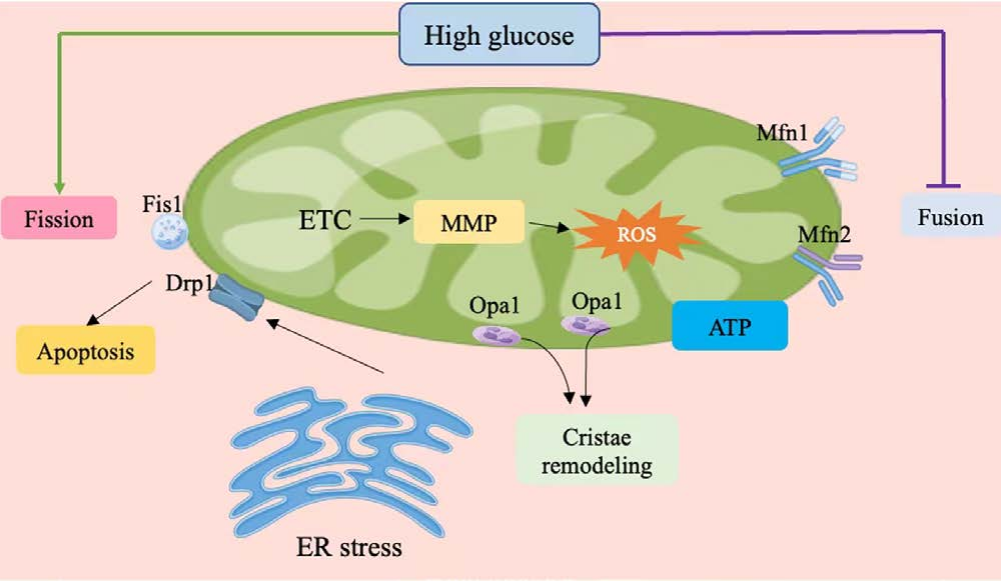
Mitochondrial dynamics. Drp1 = dynamin-related protein 1, ER = endoplasmic reticulum, ETC = electron transport chain, Fis1 = mitochondrial fission protein 1, Mfn1 = mitofusin 1, Mfn2 = mitofusin 2, MMP = mitochondrial membrane potential, Opa1 = optic atrophy protein 1.

Mitochondria are dynamic organelles that continuously undergo fusion and fission to adapt to the energy demands of the cell, ensuring an adequate distribution of mitochondria within the cellular environment.^[57]^ This process serves 2 primary functions: first, it facilitates the mixing and repair of defective mtDNA, promoting the formation of new mitochondria and effectively reducing the percentage of defective mitochondria in the cell, thereby maintaining cellular proliferation stability. Second, it allows for the redistribution of mitochondria to sites with high energy demand, which is vital for meeting the specific energy requirements of neurons.^[58]^ Mitochondrial dynamics encompass 2 key processes: mitochondrial fission and fusion. Mitochondrial fission is primarily regulated by 2 proteins: dynamin-related protein 1 (Drp1) and mitochondrial fission protein 1 (Fis1). In contrast, mitochondrial fusion is controlled by 3 major mitochondrial transmembrane proteins: mitofusin 1 (Mfn1), mitofusin 2 (Mfn2), and optic atrophy protein 1 (Opa1).^[58,59]^ Mitochondrial dynamics represent a tightly regulated cellular process, with complex molecular mechanisms involving GTPases. During mitochondrial fission, GTP hydrolysis induces conformational changes in Drp1, facilitating mitochondrial division. This process requires the assistance of Fis1.^[60]^ Excessive mitochondrial division is an early and significant event in various neurodegenerative diseases. Reports indicate that axonal swelling in the distal segments of neurons in diabetic models exhibits mitochondrial accumulation and morphological abnormalities, suggesting that mitochondrial damage may precede axonal injury.^[61]^ Consistent with these findings, another study observed elevated levels of mitochondrial fission proteins in the DRG neurons of high-fat diet (HFD)-induced DPN mice. Transmission electron microscopy revealed that mitochondrial fragmentation within DRG neurons occurred as early as 2 weeks after the onset of HFD, preceding the onset of mechanical hypersensitivity and small fiber degeneration.^[62]^ The fragmentation of mitochondria caused by excessive fission can impair cellular function.^[27,28]^ Mitochondrial fusion involves the merging of both the outer and inner mitochondrial membranes. Mfn1 and Mfn2 facilitate the fusion of the outer membrane, while Opa1 regulates the fusion of the inner membrane.^[63]^ Research indicates that DRG neurons exposed to high glucose (HG) levels exhibit mitochondrial dysfunction, fragmentation, increased Drp1 expression, and oxidative stress.^[64]^ Furthermore, hyperglycemia can stimulate the formation of Drp1/Bax complexes, thereby mediating apoptotic processes.^[65]^ Notably, significant alterations in mitochondrial dynamics have been observed in patients with T2DM, contributing to insulin resistance. Thus, mitochondrial dynamics are closely related to the progression of DPN (Fig. 2).^[66–69]^

### 3.1. Increased generation of ROS

**Figure 3.**
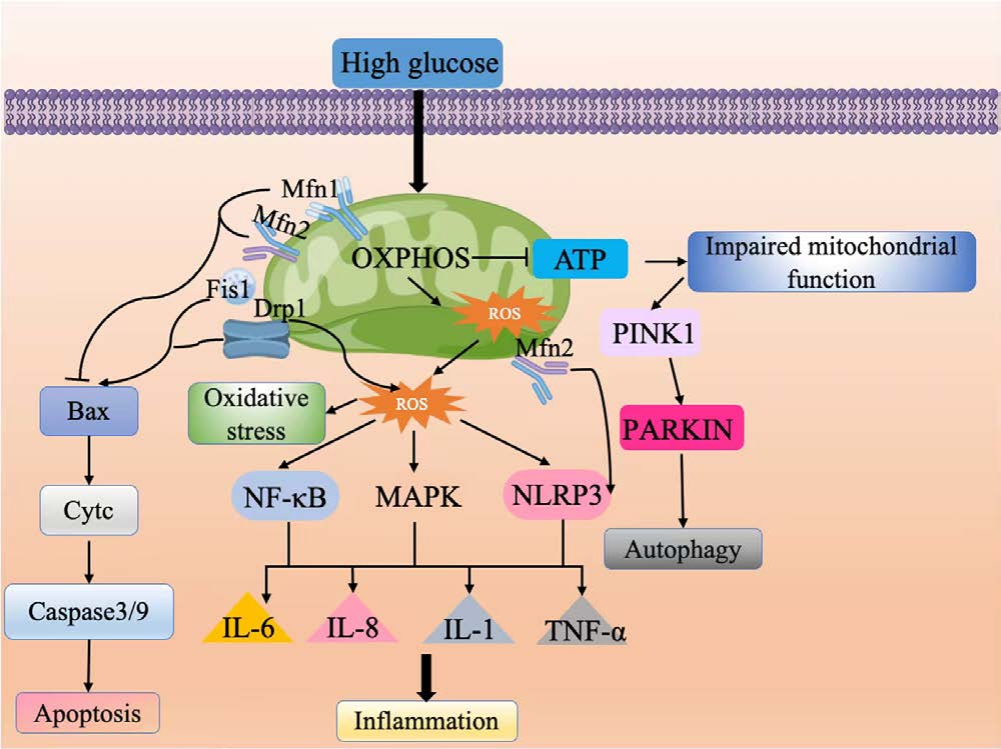
Mitochondrial dynamics mechanisms. Cytc = cytochrome C, Drp1 = dynamin-related protein 1, Fis1 = mitochondrial fission protein 1, IL = interleukin, MAPK = mitogen-activated protein kinase, Mfn1 = mitofusin 1, Mfn2 = mitofusin 2, NF-κB = nuclear factor-kappa B, NLRP3 = NOD-like receptor protein 3, OXPHOS = oxidative phosphorylation, ROS = reactive oxygen species, TNF-α = tumor necrosis factor-alpha.

Oxidative stress has garnered significant attention as a crucial pathophysiological pathway in DPN.^[70]^ The excessive production of ROS disrupts redox balance and impairs the antioxidant defense system, promoting oxidative damage associated with DPN. Mitochondria serve as the primary site for ROS generation within cells and are key targets for oxidative stress damage, playing an essential role in the regulation of metabolic imbalances in diabetes.^[71]^ Under conditions of hyperglycemic stress, DRG mitochondria, which are rich in axonal mitochondria, undergo fragmentation and functional impairment. This is characterized by increased expression of Drp1, mitochondrial fission, and dysfunction indicated by a loss of mitochondrial membrane potential (MMP), leading to insufficient energy supply and oxidative stress – considered the main cause of DRG neuronal injury in the pathogenesis of DPN.^[30,64,72,73]^ Mitochondrial respiration and oxidative phosphorylation (OXPHOS) processes are often accompanied by ROS production.^[74,75]^ Mitochondrial fusion proteins, such as Opa1 and Mfn1/2, are crucial for maintaining mtDNA integrity, stabilizing the electron transport chain complexes, and supporting overall OXPHOS capacity.^[76,77]^ Mfn2 can interact with the glycolytic enzyme pyruvate kinase, thereby enhancing mitochondrial OXPHOS while diminishing glycolysis.^[78]^ Since neurons rely solely on glycolysis for energy production, they are particularly susceptible to oxidative damage induced by increased ROS generation under hyperglycemic conditions.^[24]^ Conversely, elevated levels of Drp1-mediated mitochondrial fission decrease OXPHOS capacity, shifting the cellular bioenergetic state toward glycolysis. This shift is accompanied by a reduction in MMP and excessive ROS production.^[76,77]^ Furthermore, excessive fission severely compromises mitochondrial genes, leading to damage of mtDNA that encodes 13 polypeptides involved in OXPHOS and mitochondrial structure.^[79]^ The opening of the mitochondrial permeability transition pore also contributes to increased ROS generation. Notably, ROS production has also been reported to occur under hyperglycemic conditions in SCs and endothelial cells, resulting in MMP loss and ATP depletion, ultimately leading to neuronal loss.^[42,47,80–82]^ In addition, excessive ROS can increase the Drp1:Mfn2 ratio, promoting mitochondrial fission and elevating mitochondrial ROS, which subsequently induces endoplasmic reticulum stress and disrupts calcium homeostasis – both of which are closely associated with DPN pathogenesis.^[41,83]^ In summary, there exists a complex interplay between mitochondrial dynamics, including fission and fusion processes, and the regulation of intracellular oxidative stress, further exacerbating the pathological progression of DPN (Fig. 3).

### 3.2. Inflammatory response

Neuroinflammation is a key pathogenic factor in secondary nerve damage associated with DM. Mitochondrial dynamics can induce inflammation in DPN through various mechanisms. In microglial cells, the regulation of mitochondrial fission influences the expression of proinflammatory mediators via the activation of NF-κB and mitogen-activated protein kinase signaling pathways.^[84]^ In addition, inhibiting the mitochondrial fusion proteins Mfn1 or Mfn2 leads to increased mitochondrial fragmentation, which activates NF-κB through a toll-like receptor 9-dependent pathway.^[85]^ This fragmentation may be related to mitochondrial dysfunction caused by increased mitochondrial debris.^[86]^ The activation of NF-κB promotes the transcription of proinflammatory factors such as TNF-α, IL-1, IL-6, and IL-8.^[87]^ Notably, TNF-α typically acts as an upstream regulator of other inflammatory mediators, accelerating lipolysis, stimulating microvascular responses, inducing neuronal hypoxia, and activating downstream inflammatory factors.^[88]^ This cascade ultimately damages glial cells and neurons, leading to hypersensitivity and neuropathic pain.^[89]^ Furthermore, activated IL-6 and IL-1β directly participate in glial cytotoxicity and demyelination in DPN, resulting in endothelial dysfunction that plays a critical role in DPN pathogenesis.^[90]^ Reports indicate that skeletal muscle-specific Mfn1 knockout mice exhibit an inflammatory phenotype characterized by muscle atrophy, reduced physical activity, and a systemic inflammatory response marked by elevated IL-6 levels.^[85]^ Another study observed increased infiltration of inflammatory factors in the sciatic nerves of hyperglycemic ob/ob mice, manifesting as loss of myelinated and unmyelinated fibers along with axonal damage, highlighting significant neuropathy.^[91]^ Moreover, ROS generated by damaged mitochondria or the release of mtDNA into the cytosol are essential for NOD-like receptor protein 3 (NLRP3) inflammasome-dependent inflammatory responses.^[92]^ The activation of NLRP3 leads to the caspase-1-dependent secretion of proinflammatory cytokines such as IL-1β and IL-18.^[93]^ In specific cellular contexts, mitochondria can also serve as molecular platforms for the NLRP3 inflammasome; for instance, Mfn2 can recruit NLRP3 to assemble inflammasome complexes in response to viral infections.^[94–96]^ Multiple lines of evidence support a close association between NLRP3-mediated inflammatory responses and severe pain sensitivity in DPN.^[97–99]^ Therefore, regulating mitochondrial dynamics to mitigate neuroinflammatory damage may be significant for research aimed at slowing the pathological progression of DPN (Fig. 3).

### 3.3. Increased apoptosis

Apoptosis plays a crucial role in the pathogenesis and pathophysiology of DPN.^[100,101]^ Mitochondria, as critical regulators of apoptosis, are key players in this process. Drp1 is not only the primary protein regulating mitochondrial fission but also acts as an intrinsic factor in the mitochondria-dependent apoptotic pathway.^[102,103]^ Drp1 promotes the translocation of proapoptotic proteins, such as Bax, from the Bcl-2 family to the mitochondria, leading to increased release of cytochrome C and activation of the caspase-3/-9 signaling cascade, thereby facilitating apoptosis.^[104,105]^ Furthermore, the specific interaction between the N-terminal domain of Drp1 and Bax is enhanced during apoptosis.^[104]^ Excessive mitochondrial fission can result in dysfunctional small mitochondrial fragments, ultimately leading to cellular degeneration characterized by apoptotic features. Numerous lines of evidence indicate the presence of Drp1-mediated apoptosis in DPN.^[106]^ In studies involving DPN rat sciatic nerves and high-glucose-cultured DRG neurons, increased expression of Drp1, activation of the caspase system, and mitochondrial fragmentation have been observed, which contribute to a series of pathological processes resulting in DRG neuron damage.^[107,108]^ Notably, phosphorylation at the serine 616 residue of Drp1 may be one of the primary triggers for inducing mitochondrial fission. Furthermore, HG levels have detrimental effects on the apoptosis of SCs.^[109]^ Exposure of SCs to HG environments results in decreased MMP and ATP levels, indicating impaired mitochondrial function. This impairment activates cleaved caspase-9, which subsequently triggers the caspase-3 activation cascade, initiating apoptosis in SCs.^[110]^ In addition, other mitochondrial dynamics-related factors also participate in the apoptotic response. For instance, silencing Mfn1 or Mfn2 leads to increased mitochondrial fragmentation and heightened sensitivity to apoptotic stimuli.^[111]^ A reduction in Mfn2 disrupts mitochondrial fusion and calcium homeostasis, while simultaneously elevating levels of Bax within the mitochondria, which mediates potential neuronal apoptotic effects.^[112]^ Defects in Opa1 can also result in abnormal mitochondrial cristae and spontaneous cellular apoptosis.^[111]^ Interestingly, studies have shown that reducing Fis1 expression may suppress apoptosis to a greater extent than the reduction of Drp1.^[113]^ Notably, current research suggests that enhanced mitochondrial fission may interfere with neuronal function through apoptotic signaling pathways without necessarily leading to cell death.^[114]^ Although there is a chronic elevation of proapoptotic caspase-3 in the peripheral nerves of diabetic painful peripheral neuropathy rats, there is no evidence of sensory neuron death even after 1 year of induced neuropathy (Fig. 3).

### 3.4. Mitophagy

Autophagy is a self-clearing mechanism that maintains intracellular homeostasis by eliminating excess metabolites. Increasing evidence suggests that autophagy plays a significant role in the pathophysiology of DPN. Mitochondrial fission is crucial for mitophagy, as it facilitates the clearance of damaged mitochondria.^[115]^ Cells utilize double-membrane structures known as autophagosomes to encapsulate and recycle essential components, thereby removing defective organelles.^[116]^ Before mitophagy, mitochondrial fission generates controllable-sized fragments of individual mitochondria, making them more amenable to encapsulation.^[117]^ During the induction of mitophagy, fusion proteins Mfn1 and Mfn2 are degraded by ubiquitin-proteasome pathways, while Opa1 is targeted by the inner mitochondrial membrane zinc metalloprotease overlapping with M-AAA protease activity 1 and ATPases associated with diverse cellular activities proteases.^[118]^ Key regulators of the mitophagy process include PTEN-induced kinase 1 (PINK1), the E3 ubiquitin ligase PARKIN, ubiquitin itself, and sequestosome-1 (p62/SQSTM1). The absence of any of these proteins can lead to a failure in the selective clearance of mitochondria.^[119]^ When mitochondria become damaged, depolarization occurs, disrupting the normal proteolytic process of PINK1. This results in the accumulation of PINK1 within the mitochondria, where it phosphorylates its targets, including ubiquitin and PARKIN. Subsequently, PARKIN mediates the ubiquitination of the outer mitochondrial membrane and interacts with autophagosome-associated microtubule-associated protein 1A/1B light chain 3, ultimately leading to mitochondrial-targeted autophagy.^[120]^ Reports indicate that the induction and disruption of the autophagic process in DPN mice result in functional impairment of autophagy (Fig. 3).^[121]^

### 3.5. Insulin resistance

Insulin resistance is a common feature of T2DM. Although insulin resistance is not directly correlated with DPN, prolonged insulin resistance can lead to elevated blood glucose levels. Hyperglycemia induces vascular damage, which indirectly stimulates insulin receptors via the phosphoinositide 3-kinase/Akt pathway, accelerating cellular apoptosis.^[122]^ Several studies have indicated that gene ablation of fusion proteins alters glucose homeostasis in mice and promotes insulin resistance and obesity.^[123,124]^ Furthermore, recent evidence suggests that the knockout of Mfn1 in the liver protects mice from HFD-induced obesity and insulin resistance.^[125,126]^ This protection may be related to the increased abundance of complex I, which enhances the sensitivity of animals to the hypoglycemic effects of metformin.^[125]^ Research has also found that Mfn2-deficient mice are prone to insulin resistance, aligning with previous observations of Mfn2 suppression in obesity and T2DM.^[124,127,128]^ In addition, oxidative stress has been implicated in the pathogenesis of T2DM, particularly concerning changes in β-cell MMP. These alterations in β cells may be closely linked to changes in mitochondrial dynamics, leading to impaired insulin secretion in response to glucose stimulation.^[129,130]^ In T2DM, the processes of β-cell fusion and fission undergo continuous modification; studies indicate that elevated blood glucose and palmitate levels reduce fusion events and inhibit mitochondrial oxygen consumption.^[131,132]^ Drp1-mediated mitochondrial fission may be a contributing factor to insulin resistance. Another study reported that in C2C12 cells precultured with palmitate, Drp1-mediated fission resulted in mitochondrial fragmentation, loss of MMP, increased oxidative stress, decreased ATP levels, and reduced insulin-mediated glucose uptake. However, Drp1 knockdown experiments reversed these changes.^[133]^ Abnormal glucose homeostasis may arise as a consequence of altered mitochondrial dynamics. In recent years, the beneficial effects of exercise on DPN have been extensively studied.^[134–136]^ Research demonstrates that exercise stabilizes mitochondrial dynamics by decreasing Drp1 expression and increasing the expression of Mfn1 and Mfn2, thus promoting fatty acid oxidation and improving insulin sensitivity.^[137]^ Stabilizing mitochondrial dynamics may help ameliorate insulin resistance and improve glycemic control, potentially alleviating the symptoms of DPN (Fig. 3).

## 4 . Signaling pathways related to mitochondrial dynamics

### 4.1. Adenosine monophosphate-activated protein kinase signaling pathway

Adenosine monophosphate-activated protein kinase (AMPK) is regarded as a crucial molecule in mitochondrial quality control and serves as a master regulator of glucose and lipid metabolism.^[138–140]^ AMPK can sense metabolic stress, integrating various physiological signals to restore energy balance.^[141,142]^ It plays a pivotal role in regulating cell survival or death under pathological stresses such as hypoxia, osmotic stress, and oxidative stress.^[143]^ Consequently, AMPK is key to the alterations in cellular metabolism induced by hyperglycemia.^[144]^ AMPK is composed of 2 regulatory β and γ subunits, along with a catalytic α subunit.^[145]^ Upon activation, AMPK promotes mitochondrial elongation/fusion and proliferation, resulting in increased ATP production and reduced ROS, thereby enhancing cell survival.^[138,146]^ Reports indicate that long-term hyperglycemia associated with nutrient excess may lead to the shutdown of AMPK and/or silent information regulator T1 (SIRT1) signaling pathways, which subsequently impairs the activity of peroxisome proliferator-activated receptor gamma coactivator 1-alpha (PGC-1α) and reduces mitochondrial activity.^[147]^ In rat DRG cells stimulated by HG, AMPK expression is suppressed.^[99]^ Similarly, reduced levels of phosphorylated AMPK have been observed in the nerves of diabetic mice.^[148,149]^ AMPK is directly involved in regulating mitochondrial dynamics, with the key outer mitochondrial membrane receptor protein mitochondrial fission factor acting as a substrate for AMPK. The phosphorylation of mitochondrial fission factor by AMPK is essential for mitochondrial fission.^[150]^ Furthermore, AMPK can indirectly regulate mitochondrial homeostasis through targeting transcriptional regulators. Activation of AMPK leads to the direct phosphorylation of PGC-1α at threonine 177 and serine 538, which is critical for the activation of PGC-1α-dependent transcription.^[151]^ The activation of PGC-1α stimulates the expression of Mfn2 and enhances mitochondrial fusion activity.^[152]^ In DPN, PGC-1α activates transcription factors such as Nrf2 and regulates mitochondrial degeneration, with Nrf2 being considered a central coordinator of mitochondrial dynamics.^[153–156]^ In addition, another study confirmed that the activation of the AMPK signaling pathway promotes the stability of Opa1, whereas its blockade inhibits Opa1 expression and compromises its cardioprotective effects.^[157]^ Through modulation of Opa1-related mitochondrial activity and dynamics, AMPK participates in the molecular mechanisms underlying the prevention of aging, obesity, and drug-induced liver injury.^[138,158]^ In conclusion, targeting the AMPK signaling pathway may represent a promising therapeutic strategy for DPN.

### 4.2. p53 signaling pathway

The p53 tumor suppressor gene plays a critical role in inducing and maintaining cellular senescence. Evidence suggests that mitochondrial dynamics, specifically fission and fusion processes, are regulated by p53. Research has shown that p53 is upregulated in damaged neurons of DPN rat models and exhibits neurotoxic effects in models of neurodegenerative diseases.^[159]^ Notably, Mfn2 has been identified as a direct downstream target of p53.^[115,160]^ Furthermore, p53 can induce excessive mitochondrial fission mediated by Drp1, leading to severe damage to the mitochondrial genome. This damage results in impairments to mtDNA that encodes proteins essential for OXPHOS and mitochondrial structure, ultimately increasing the production of ROS and exacerbating neuronal oxidative injury.^[161]^ High concentrations of glucose have been shown to induce apoptosis in hippocampal neurons by downregulating SIRT1 and increasing p53 acetylation.^[162]^ Another study reported that astragaloside IV could reduce the occurrence of mitochondrial-dependent apoptosis in DRG neurons of DPN rats by modulating the SIRT1/p53 pathway. This intervention enhanced the activity of mitochondrial electron transport chain complexes and improved MMP, alleviating the pathological symptoms of DPN.^[163]^ These findings indicate that p53 influences the progression of DPN through the regulation of mitochondrial dynamics. A deeper understanding of this mechanism may provide a theoretical basis for the development of new therapeutic strategies.

### 4.3. AGEs/receptor for AGEs signaling pathway

Nonenzymatic reactions produce AGEs, which contribute to the cross-linking of intracellular and extracellular proteins, as well as necessary protein modifications. AGEs are deposited in almost every part of neural tissue, and their accumulation is associated with a reduction in the density of myelinated nerve fibers.^[164]^ Furthermore, AGEs interact with cell surface receptors, primarily the receptor for AGEs (RAGE), activating downstream signaling cascades that lead to chronic inflammatory responses and neuronal damage, thereby promoting the development of DPN.^[165,166]^ Notably, the Janus kinase 2–signal transducer and activator of transcription 3 pathway in microglia has been implicated in neuroinflammation induced by Drp1-dependent mitochondrial fission,^[167]^ suggesting a potential interplay between AGEs/RAGE signaling and mitochondrial dysfunction in DPN pathogenesis. The AGE/RAGE signaling pathway is also crucial for glucose metabolism, linking it to the pathophysiology of DM and its associated microvascular complications.^[165,168,169]^ Studies indicate that elevated levels of AGEs can damage pancreatic β cells, resulting in reduced insulin secretion. This impairment may be related to AGEs disrupting mitochondrial function and dynamics in β cells, leading to an imbalance in mitochondrial fusion and fission processes, as well as mitophagy.^[129]^ Future research should further explore how the modulation of mitochondrial dynamics and metabolic pathways in DM can intervene in these pathophysiological processes, providing new strategies and targets for treatment.

## 5. Therapeutic approaches

Mitochondria play several crucial roles in cellular functions, including the production of ATP and ROS, as well as the regulation of cell survival and apoptosis. Moreover, oxidative stress and mitochondrial dysfunction are closely associated with DPN. For these reasons, mitochondria represent a key therapeutic target. Clinical trials involving antioxidants have reported only modest alleviation of symptoms in patients with various forms of painful peripheral neuropathy, indicating that therapeutic interventions targeting upstream ROS generation may be more effective.^[170,171]^ Mitochondrial fission may be a prerequisite for excessive ROS production; thus, inhibiting mitochondrial fragmentation might be beneficial in alleviating ROS-dependent pain symptoms associated with neuropathy.^[172]^ Mitochondrial fission is a critical target in the treatment of metabolic diseases. Mitochondrial division inhibitor 1 (Mdivi-1) is a compound that inhibits the GTPase activity of Drp1. Ferrari et al demonstrated that intradermal injection of Mdivi-1 significantly alleviated mechanical allodynia induced by oxaliplatin in painful peripheral neuropathy models.^[173,174]^ In addition, Mdivi-1 significantly improved mitochondrial distribution disturbances in spinal cord cells under acute high-glucose conditions.^[175]^ By modulating Drp1 activity, Mdivi-1 enhances mitochondrial function and reduces ROS production in the presence of excess palmitic acid.^[133]^ Under hyperglycemic conditions in diabetic animal models, Mdivi-1 has been shown to reduce atherosclerosis, inflammation, and oxidative stress, thereby exerting beneficial effects.^[176]^ However, other studies suggest that prolonged use of this compound may impair mitochondrial function, indicating its effectiveness is limited to short-term applications.^[177]^ It is also important to note that Mdivi-1 exists as 2 enantiomers, which could potentially affect its biological activity differently, though it remains unclear whether these isomers have distinct effects. The observed effects of Mdivi-1 may also depend on experimental conditions, and further research is needed to clarify how this compound influences mitochondrial dynamics and function, especially in terms of its interaction with Drp1 and its potential off-target effects on the electron transport chain.^[178]^ Wu et al^[107]^ explored the protective effects of glimepiride on sciatic nerve injury in streptozotocin (STZ)-induced diabetic rats. They found that glimepiride improved nerve conduction velocity and remyelination of the sciatic nerve, possibly through the modulation of Drp1-mediated oxidative stress and apoptosis, providing protection against DPN. Quercetin, a natural AMPK activator, has been shown to correct oxidative stress, reduced ATP production, and altered mitochondrial morphology in the sciatic nerve and SCs affected by DPN. This neuroprotective effect is primarily associated with the activation of the AMPK/PGC-1α pathway, which protects mitochondrial activity.^[179]^ Melatonin also plays a significant role in regulating mitochondrial fission and fusion. It has been reported that melatonin treatment reduces Drp1 expression in STZ-induced diabetic mice, thereby inhibiting mitochondrial fragmentation, reducing oxidative stress, and decreasing cardiomyocyte apoptosis. These effects improve mitochondrial function and cardiac performance, potentially mediated by melatonin’s ability to downregulate Drp1-mediated mitochondrial fission in a SIRT1/PGC-1α-dependent manner, contributing to its cardioprotective actions.^[180]^

In addition, various traditional Chinese medicines may target mitochondrial dynamics to treat DPN. For example, Zhu et al^[181]^ utilized a traditional herbal formula, Tangluoning, for the treatment of DPN. They found that Tangluoning increased the expression of mitochondrial fusion-related proteins Mfn1, Mfn2, and Opal, while simultaneously inhibiting the expression and phosphorylation of Drp1, a protein associated with mitochondrial fission. This resulted in a significant improvement in mitochondrial function in DPN rats, thereby exerting a protective effect against neuronal damage. Another compound of interest is astragaloside IV (AS-IV), a bioactive saponin isolated from the root of Astragalus membranaceus. Ben et al^[163]^ investigated the beneficial effects of AS-IV on DRGs in STZ and high-carbohydrate/high-fat diet-induced diabetic rats. Their study demonstrated that AS-IV significantly alleviated hyperalgesia and increased nerve conduction velocity. Furthermore, AS-IV reduced mitochondrial-dependent apoptosis through the modulation of the SIRT1/p53 pathway, thereby decreasing Drp1-induced excessive mitochondrial fission. These findings suggest that AS-IV may serve as a promising candidate for the treatment of DPN.

## 6. Discussion

DPN stands as one of the most prevalent complications associated with DM, contributing to over half of all nontraumatic amputations. Despite the availability of various pharmacological treatments and therapeutic strategies aimed at managing pain and halting symptom progression, an effective and universally accepted treatment remains elusive. In the quest to elucidate the underlying mechanisms of DPN, researchers have identified a critical aspect of mitochondrial homeostasis: the balance between mitochondrial fission and fusion processes. Disruption of this delicate balance can lead to excessive mitochondrial fragmentation, resulting in mitochondrial dysfunction that initiates a cascade of pathological processes. These processes include metabolic dysregulation, apoptosis, inflammation, and oxidative stress, all of which are likely pivotal in the onset and progression of DPN. Targeting mitochondrial dynamics to preserve mitochondrial integrity and function emerges as a promising therapeutic strategy for DPN. Interventions designed to restore the equilibrium between fission and fusion may mitigate the detrimental effects of hyperglycemia, thereby enhancing neuronal health. Future research should prioritize the development of innovative compounds or therapies that specifically promote mitochondrial fusion while inhibiting excessive fission. Moreover, investigating the interplay between mitochondrial dynamics and other cellular pathways implicated in neuropathic pain could reveal new therapeutic targets. A comprehensive understanding of these mechanisms is essential for developing effective treatments for DPN, ultimately improving patient outcomes, and reducing the incidence of severe complications. Through continued exploration of mitochondrial dynamics and their role in DPN, we may pave the way for novel interventions that significantly enhance the quality of life for patients suffering from this debilitating condition.

## Author contributions

**Writing – original draft:** Honghai Yu, Cunqing Yang.

**Writing – review & editing:** Honghai Yu, Cunqing Yang.

**Funding acquisition:** Guoqiang Wang, Xiuge Wang.

**Supervision:** Guoqiang Wang, Xiuge Wang.

## References

[1] International Diabetes Federation. IDF Diabetes Atlas. 8th ed. International Diabetes Federation. 2017:905–911.

[2] Huang D Refaat M Mohammedi K Jayyousi A Al Suwaidi J Abi Khalil C. Macrovascular complications in patients with diabetes and prediabetes. Biomed Res Int. 2017;2017:7839101. 29238721 10.1155/2017/7839101 PMC5697393

[3] Valencia WM Florez H. How to prevent the microvascular complications of type 2 diabetes beyond glucose control. BMJ. 2017;356:i6505. 28096078 10.1136/bmj.i6505

[4] Stratton IM Adler AI Neil HAW. Association of glycaemia with macrovascular and microvascular complications of type 2 diabetes (UKPDS 35): prospective observational study. BMJ. 2000;321:405–12. 10938048 10.1136/bmj.321.7258.405 PMC27454

[5] Prompers L Schaper N Apelqvist J. Prediction of outcome in individuals with diabetic foot ulcers: focus on the differences between individuals with and without peripheral arterial disease. The EURODIALE Study. Diabetologia. 2008;51:747–55. 18297261 10.1007/s00125-008-0940-0 PMC2292424

[6] Albers JW Pop-Busui R. Diabetic neuropathy: mechanisms, emerging treatments, and subtypes. Curr Neurol Neurosci Rep. 2014;14:1–11. 10.1007/s11910-014-0473-5 PMC5084622 24954624

[7] Singh R Kishore L Kaur N. Diabetic peripheral neuropathy: current perspective and future directions. Pharmacol Res. 2014;80:21–35. 24373831 10.1016/j.phrs.2013.12.005

[8] Bahnasy WS El-Heneedy YAE El-Seidy EAS Labib NAA Ibrahim ISE. Sleep disturbances in diabetic peripheral neuropathy patients: a clinical and polysomnographic study. Egypt J Neurol Psychiatr Neurosurg. 2018;54:1–7. 30237691 10.1186/s41983-018-0024-0 PMC6133053

[9] Gore M Brandenburg NA Dukes E Hoffman DL Tai K-S Stacey B. Pain severity in diabetic peripheral neuropathy is associated with patient functioning, symptom levels of anxiety and depression, and sleep. J Pain Symptom Manage. 2005;30:374–85. 16256902 10.1016/j.jpainsymman.2005.04.009

[10] Meyer‐Hamme G Friedemann T Greten J Gerloff C Schroeder S. Electrophysiologically verified effects of acupuncture on diabetic peripheral neuropathy in type 2 diabetes: the randomized, partially double-blinded, controlled ACUDIN trial. J Diabetes. 2021;13:469–81. 33150711 10.1111/1753-0407.13130

[11] Feldman EL Callaghan BC Pop-Busui R. Diabetic neuropathy. Nat Rev Dis Primers. 2019;5:41. 31197153 10.1038/s41572-019-0092-1

[12] Pop-Busui R Herman WH Feldman EL; DCCT/EDIC Research Group. DCCT and EDIC studies in type 1 diabetes: lessons for diabetic neuropathy regarding metabolic memory and natural history. Curr Diab Rep. 2010;10:276–82. 20464532 10.1007/s11892-010-0120-8 PMC3608672

[13] Malik RA. Why are there no good treatments for diabetic neuropathy? Lancet Diabetes Endocrinol. 2014;2:607–9. 24746878 10.1016/S2213-8587(14)70067-1

[14] Vincent AM Callaghan BC Smith AL Feldman EL. Diabetic neuropathy: cellular mechanisms as therapeutic targets. Nat Rev Neurol. 2011;7:573–83. 21912405 10.1038/nrneurol.2011.137

[15] Morales-Vidal S Morgan C McCoyd M Hornik A. Diabetic peripheral neuropathy and the management of diabetic peripheral neuropathic pain. Postgrad Med. 2012;124:145–53. 22913903 10.3810/pgm.2012.07.2576

[16] Malik RA Newrick PG Sharma AK. Microangiopathy in human diabetic neuropathy: relationship between capillary abnormalities and the severity of neuropathy. Diabetologia. 1989;32:92–102. 2721843 10.1007/BF00505180

[17] Baum P Toyka KV Blüher M Kosacka J Nowicki M. Inflammatory mechanisms in the pathophysiology of diabetic peripheral neuropathy (DN) – new aspects. Int J Mol Sci . 2021;22:10835. 34639176 10.3390/ijms221910835 PMC8509236

[18] Feldman EL Callaghan BC Pop-Busui R. Diabetic neuropathy. Nat Rev Dis Primers. 2019;5:1–18. 31197153 10.1038/s41572-019-0092-1

[19] Dejgaard A. Pathophysiology and treatment of diabetic neuropathy. Diabet Med. 1998;15:97–112. 9507909 10.1002/(SICI)1096-9136(199802)15:2<97::AID-DIA523>3.0.CO;2-5

[20] Li J Guan R Pan L. Mechanism of Schwann cells in diabetic peripheral neuropathy: a review. Medicine (Baltimore). 2023;102:e32653. 36607875 10.1097/MD.0000000000032653 PMC9829292

[21] Elafros MA Andersen H Bennett DL. Towards prevention of diabetic peripheral neuropathy: clinical presentation, pathogenesis, and new treatments. Lancet Neurol. 2022;21:922–36. 36115364 10.1016/S1474-4422(22)00188-0 PMC10112836

[22] Partanen J Niskanen L Lehtinen J Mervaala E Siitonen O Uusitupa M. Natural history of peripheral neuropathy in patients with non-insulin-dependent diabetes mellitus. N Engl J Med. 1995;333:89–94. 7777034 10.1056/NEJM199507133330203

[23] Brownlee M Hirsch IB. Glycemic variability: a hemoglobin A1c–independent risk factor for diabetic complications. JAMA. 2006;295:1707–8. 16609094 10.1001/jama.295.14.1707

[24] Leinninger GM Edwards JL Lipshaw MJ Feldman EL. Mechanisms of disease: mitochondria as new therapeutic targets in diabetic neuropathy. Nat Clin Pract Neurol. 2006;2:620–8. 17057749 10.1038/ncpneuro0320

[25] Pinti MV Fink GK Hathaway QA Durr AJ Kunovac A Hollander JM. Mitochondrial dysfunction in type 2 diabetes mellitus: an organ-based analysis. Am J Physiol Endocrinol Metab. 2019;316:E268–85. 30601700 10.1152/ajpendo.00314.2018 PMC6397358

[26] Chandrasekaran K Anjaneyulu M Choi J. Role of mitochondria in diabetic peripheral neuropathy: Influencing the NAD+-dependent SIRT1–PGC-1α–TFAM pathway. Int Rev Neurobiol. 2019;145:177–209. 31208524 10.1016/bs.irn.2019.04.002 PMC6590704

[27] Parone PA Da Cruz S Tondera D. Preventing mitochondrial fission impairs mitochondrial function and leads to loss of mitochondrial DNA. PLoS One. 2008;3:e3257. 18806874 10.1371/journal.pone.0003257 PMC2532749

[28] Wang X Su B Lee H-g. Impaired balance of mitochondrial fission and fusion in Alzheimer’s disease. J Neurosci. 2009;29:9090–103. 19605646 10.1523/JNEUROSCI.1357-09.2009 PMC2735241

[29] Russell JW Sullivan KA Windebank AJ Herrmann DN Feldman EL. Neurons undergo apoptosis in animal and cell culture models of diabetes. Neurobiol Dis. 1999;6:347–63. 10527803 10.1006/nbdi.1999.0254

[30] Vincent AM Edwards JL McLean LL. Mitochondrial biogenesis and fission in axons in cell culture and animal models of diabetic neuropathy. Acta Neuropathol. 2010;120:477–89. 20473509 10.1007/s00401-010-0697-7 PMC4254759

[31] Eid SA Rumora AE Beirowski B. New perspectives in diabetic neuropathy. Neuron. 2023;111:2623–41. 37263266 10.1016/j.neuron.2023.05.003 PMC10525009

[32] Said G Slama G Selva J. Progressive centripetal degeneration of axons in small fibre diabetic polyneuropathy: a clinical and pathological study. Brain. 1983;106 (Pt 4):791–807. 6652463 10.1093/brain/106.4.791

[33] Garcia‐Perez E Schönberger T Sumalla M. Behavioural, morphological and electrophysiological assessment of the effects of type 2 diabetes mellitus on large and small nerve fibres in Zucker diabetic fatty, Zucker lean and Wistar rats. Eur J Pain. 2018;22:1457–72. 29676840 10.1002/ejp.1235

[34] Malik RA Tesfaye S Newrick PG. Sural nerve pathology in diabetic patients with minimal but progressive neuropathy. Diabetologia. 2005;48:578–85. 15729579 10.1007/s00125-004-1663-5

[35] Smith S Normahani P Lane T Hohenschurz-Schmidt D Oliver N Davies AH. Pathogenesis of distal symmetrical polyneuropathy in diabetes. Life (Basel, Switzerland). 2022;12:1074. 35888162 10.3390/life12071074 PMC9319251

[36] Rosenberger DC Blechschmidt V Timmerman H Wolff A Treede R-D. Challenges of neuropathic pain: focus on diabetic neuropathy. J Neural Transmission (Vienna, Austria :1996). 2020;127:589–624. 10.1007/s00702-020-02145-7 PMC7148276 32036431

[37] Scholz J Broom DC Youn D-H. Blocking caspase activity prevents transsynaptic neuronal apoptosis and the loss of inhibition in lamina II of the dorsal horn after peripheral nerve injury. J Neurosci. 2005;25:7317–23. 16093381 10.1523/JNEUROSCI.1526-05.2005 PMC6725303

[38] DiAntonio A. Axon degeneration: mechanistic insights lead to therapeutic opportunities for the prevention and treatment of peripheral neuropathy. Pain. 2019;160(Suppl 1):S17–22. 31008845 10.1097/j.pain.0000000000001528 PMC6481657

[39] Modrak M Hassan Talukder MA Gurgenashvili K. Peripheral nerve injury and myelination: potential therapeutic strategies. J Neurosci Res. 2020;98:780–95. 31608497 10.1002/jnr.24538 PMC7072007

[40] Saleh A Chowdhury SKR Smith DR. Ciliary neurotrophic factor activates NF-κB to enhance mitochondrial bioenergetics and prevent neuropathy in sensory neurons of streptozotocin-induced diabetic rodents. Neuropharmacology. 2013;65:65–73. 23022047 10.1016/j.neuropharm.2012.09.015 PMC3521091

[41] O’Brien PD Hinder LM Sakowski SA Feldman EL. ER stress in diabetic peripheral neuropathy: a new therapeutic target. Antioxid Redox Signal. 2014;21:621–33. 24382087 10.1089/ars.2013.5807

[42] Vincent AM Mclean LL Backus C Feldman EL. Short-term hyperglycemia produces oxidative damage and apoptosis in neurons. FASEB J. 2005;19:1–24. 15677696 10.1096/fj.04-2513fje

[43] Dunnigan SK Ebadi H Breiner A. Conduction slowing in diabetic sensorimotor polyneuropathy. Diabetes Care. 2013;36:3684–90. 24026550 10.2337/dc13-0746 PMC3816879

[44] Pang L Lian X Liu H. Understanding diabetic neuropathy: focus on oxidative stress. Oxid Med Cell Longevity. 2020;2020:9524635. 10.1155/2020/9524635 PMC7422494 32832011

[45] Gonçalves NP Vægter CB Andersen H Østergaard L Calcutt NA Jensen TS. Schwann cell interactions with axons and microvessels in diabetic neuropathy. Nat Rev Neurol. 2017;13:135–47. 28134254 10.1038/nrneurol.2016.201 PMC7391875

[46] Zhang C-H Lv X Du W. The Akt/mTOR cascade mediates high glucose-induced reductions in BDNF via DNMT1 in Schwann cells in diabetic peripheral neuropathy. Exp Cell Res. 2019;383:111502. 31323191 10.1016/j.yexcr.2019.111502

[47] Brownlee M. Biochemistry and molecular cell biology of diabetic complications. Nature. 2001;414:813–20. 11742414 10.1038/414813a

[48] Clements RS Jr Vourganti B Kuba T Oh SJ Darnell B. Dietary myo-inositol intake and peripheral nerve function in diabetic neuropathy. Metabolism. 1979;28:477–83. 262775 10.1016/0026-0495(79)90060-x

[49] Salway J Finnegan J Barnett D Whitehead L Karunanayaka A Payne RB. Effect of myo-inositol on peripheral-nerve function in diabetes. Lancet. 1978;312:1282–4. 10.1016/s0140-6736(78)92043-3 82784

[50] Nukada H. Ischemia and diabetic neuropathy. Handb Clin Neurol. 2014;126:469–87. 25410240 10.1016/B978-0-444-53480-4.00023-0

[51] Lim TK Shi XQ Johnson JM. Peripheral nerve injury induces persistent vascular dysfunction and endoneurial hypoxia, contributing to the genesis of neuropathic pain. J Neurosci. 2015;35:3346–59. 25716835 10.1523/JNEUROSCI.4040-14.2015 PMC6605560

[52] Paterson K Lolignier S Wood JN McMahon SB Bennett DLH. Botulinum toxin – a treatment reduces human mechanical pain sensitivity and mechanotransduction. Ann Neurol. 2014;75:591–6. 24550077 10.1002/ana.24122 PMC4112716

[53] Newrick PG Wilson AJ Jakubowski J. Sural nerve oxygen tension in diabetes. Br Med J (Clin Res Ed). 1986;293:1053–4. 10.1136/bmj.293.6554.1053 PMC1341910 3094772

[54] Trachootham D Lu W Ogasawara MA Nilsa RV Huang P. Redox regulation of cell survival. Antioxid Redox Signal. 2008;10:1343–74. 18522489 10.1089/ars.2007.1957 PMC2932530

[55] Poulsen HE Specht E Broedbaek K. RNA modifications by oxidation: a novel disease mechanism? Free Radic Biol Med. 2012;52:1353–61. 22306201 10.1016/j.freeradbiomed.2012.01.009

[56] Giacco F Brownlee M. Oxidative stress and diabetic complications. Circ Res. 2010;107:1058–70. 21030723 10.1161/CIRCRESAHA.110.223545 PMC2996922

[57] Chan DC. Mitochondria: dynamic organelles in disease, aging, and development. Cell. 2006;125:1241–52. 16814712 10.1016/j.cell.2006.06.010

[58] Knott AB Perkins G Schwarzenbacher R Bossy-Wetzel E. Mitochondrial fragmentation in neurodegeneration. Nat Rev Neurosci. 2008;9:505–18. 18568013 10.1038/nrn2417 PMC2711514

[59] Smirnova E Griparic L Shurland D-L van der Bliek AM. Dynamin-related protein Drp1 is required for mitochondrial division in mammalian cells. Mol Biol Cell. 2001;12:2245–56. 11514614 10.1091/mbc.12.8.2245 PMC58592

[60] Ingerman E Perkins EM Marino M. Dnm1 forms spirals that are structurally tailored to fit mitochondria. J Cell Biol. 2005;170:1021–7. 16186251 10.1083/jcb.200506078 PMC2171542

[61] Ebenezer GJ McArthur JC Thomas D. Denervation of skin in neuropathies: the sequence of axonal and Schwann cell changes in skin biopsies. Brain. 2007;130:2703–14. 17898011 10.1093/brain/awm199

[62] George DS Hackelberg S Jayaraj ND. Mitochondrial calcium uniporter deletion prevents painful diabetic neuropathy by restoring mitochondrial morphology and dynamics. Pain. 2022;163:560–78. 34232927 10.1097/j.pain.0000000000002391 PMC8720329

[63] Ishihara N Eura Y Mihara K. Mitofusin 1 and 2 play distinct roles in mitochondrial fusion reactions via GTPase activity. J Cell Sci. 2004;117:6535–46. 15572413 10.1242/jcs.01565

[64] Leinninger GM Backus C Sastry AM Yi Y-B Wang C-W Feldman EL. Mitochondria in DRG neurons undergo hyperglycemic mediated injury through Bim, Bax and the fission protein Drp1. Neurobiol Dis. 2006;23:11–22. 16684605 10.1016/j.nbd.2006.01.017

[65] Karbowski M Lee Y-J Gaume B. Spatial and temporal association of Bax with mitochondrial fission sites, Drp1, and Mfn2 during apoptosis. J Cell Biol. 2002;159:931–8. 12499352 10.1083/jcb.200209124 PMC2173996

[66] Rovira-Llopis S Bañuls C Diaz-Morales N Hernandez-Mijares A Rocha M Victor VM. Mitochondrial dynamics in type 2 diabetes: pathophysiological implications. Redox Biol. 2017;11:637–45. 28131082 10.1016/j.redox.2017.01.013 PMC5284490

[67] Lin H-Y Weng S-W Chang Y-H. The causal role of mitochondrial dynamics in regulating insulin resistance in diabetes: link through mitochondrial reactive oxygen species. Oxid Med Cell Longevity. 2018;2018:7514383. 10.1155/2018/7514383 PMC6186363 30363990

[68] Dubé JJ Collyer ML Trant S. Decreased mitochondrial dynamics is associated with insulin resistance, metabolic rate, and fitness in African Americans. J Clin Endocrinol Metab. 2020;105:1210–20. 31833547 10.1210/clinem/dgz272 PMC7067552

[69] Houzelle A Jörgensen JA Schaart G. Human skeletal muscle mitochondrial dynamics in relation to oxidative capacity and insulin sensitivity. Diabetologia. 2021;64:424–36. 33258025 10.1007/s00125-020-05335-w PMC7801361

[70] Lin Q Li K Chen Y. Oxidative stress in diabetic peripheral neuropathy: pathway and mechanism-based treatment. Mol Neurobiol. 2023;60:4574–94. 37115404 10.1007/s12035-023-03342-7

[71] Yu T Robotham JL Yoon Y. Increased production of reactive oxygen species in hyperglycemic conditions requires dynamic change of mitochondrial morphology. Proc Natl Acad Sci USA. 2006;103:2653–8. 16477035 10.1073/pnas.0511154103 PMC1413838

[72] Senyilmaz D Virtue S Xu X. Regulation of mitochondrial morphology and function by stearoylation of TFR1. Nature. 2015;525:124–8. 26214738 10.1038/nature14601 PMC4561519

[73] Patergnani S Morciano G Carinci M Leo S Pinton P Rimessi A. The “mitochondrial stress responses”: the “Dr. Jekyll and Mr. Hyde” of neuronal disorders. Neural Regener Res. 2022;17:2563–75. 10.4103/1673-5374.339473 PMC9165365 35662183

[74] Smeitink JA Zeviani M Turnbull DM Jacobs HT. Mitochondrial medicine: a metabolic perspective on the pathology of oxidative phosphorylation disorders. Cell Metab. 2006;3:9–13. 16399500 10.1016/j.cmet.2005.12.001

[75] Rossmann MP Dubois SM Agarwal S Zon LI. Mitochondrial function in development and disease. Dis Model Mech. 2021;14:dmm048912. 34114603 10.1242/dmm.048912 PMC8214736

[76] Yao C-H Wang R Wang Y Kung C-P Weber JD Patti GJ. Mitochondrial fusion supports increased oxidative phosphorylation during cell proliferation. Elife. 2019;8:e41351. 30694178 10.7554/eLife.41351 PMC6351101

[77] Punter KB Chu C Chan EY. Mitochondrial dynamics and oxidative phosphorylation as critical targets in cancer. Endocr Relat Cancer. 2022;30:e220229. 36356297 10.1530/ERC-22-0229

[78] Li T Han J Jia L Hu X Chen L Wang Y. PKM2 coordinates glycolysis with mitochondrial fusion and oxidative phosphorylation. Protein Cell. 2019;10:583–94. 30887444 10.1007/s13238-019-0618-z PMC6626593

[79] Zhou H Hu S Jin Q. Mff-dependent mitochondrial fission contributes to the pathogenesis of cardiac microvasculature ischemia/reperfusion injury via induction of mROS-mediated cardiolipin oxidation and HK 2/VDAC 1 disassociation-involved mPTP opening. J Am Heart Assoc. 2017;6:e005328. 28288978 10.1161/JAHA.116.005328 PMC5524036

[80] Russell JW Golovoy D Vincent AM. High glucose-induced oxidative stress and mitochondrial dysfunction in neurons. FASEB J. 2002;16:1738–48. 12409316 10.1096/fj.01-1027com

[81] Brownlee M. The pathobiology of diabetic complications: a unifying mechanism. Diabetes. 2005;54:1615–25. 15919781 10.2337/diabetes.54.6.1615

[82] Boulton AJ Vinik AI Arezzo JC; American Diabetes Association. Diabetic neuropathies: a statement by the American Diabetes Association. Diabetes Care. 2005;28:956–62. 15793206 10.2337/diacare.28.4.956

[83] Cooper ID Brookler KH Kyriakidou Y Elliott BT Crofts CAP. Metabolic phenotypes and step by step evolution of type 2 diabetes: a new paradigm. Biomedicines. 2021;9:800. 34356863 10.3390/biomedicines9070800 PMC8301386

[84] Park J Choi H Min JS. Mitochondrial dynamics modulate the expression of pro-inflammatory mediators in microglial cells. J Neurochem. 2013;127:221–32. 23815397 10.1111/jnc.12361

[85] Irazoki A Gordaliza-Alaguero I Frank E. Disruption of mitochondrial dynamics triggers muscle inflammation through interorganellar contacts and mitochondrial DNA mislocation. Nat Commun. 2023;14:108. 36609505 10.1038/s41467-022-35732-1 PMC9822926

[86] Zhang X Huang W Shao Q. Drp1, a potential therapeutic target for Parkinson’s disease, is involved in olfactory bulb pathological alteration in the rotenone-induced rat model. Toxicol Lett. 2020;325:1–13. 32088201 10.1016/j.toxlet.2020.02.009

[87] Schreck R Rieber P Baeuerle PA. Reactive oxygen intermediates as apparently widely used messengers in the activation of the NF-kappa B transcription factor and HIV‐1. EMBO J. 1991;10:2247–58. 2065663 10.1002/j.1460-2075.1991.tb07761.x PMC452914

[88] Subedi L Lee SE Madiha S. Phytochemicals against TNF α-mediated neuroinflammatory diseases. Int J Mol Sci . 2020;21:764. 31991572 10.3390/ijms21030764 PMC7037901

[89] Kawasaki Y Zhang L Cheng J-K Ji R-R. Cytokine mechanisms of central sensitization: distinct and overlapping role of interleukin-1β, interleukin-6, and tumor necrosis factor-α in regulating synaptic and neuronal activity in the superficial spinal cord. J Neurosci. 2008;28:5189–94. 18480275 10.1523/JNEUROSCI.3338-07.2008 PMC2408767

[90] Godambe SA Knapp KM Meals EA English BK. Role of vav1 in the lipopolysaccharide-mediated upregulation of inducible nitric oxide synthase production and nuclear factor for interleukin-6 expression activity in murine macrophages. Clin Diagn Lab Immunol. 2004;11:525–31. 15138177 10.1128/CDLI.11.3.525-531.2004 PMC404562

[91] Kosacka J Nowicki M Klöting N. COMP-angiopoietin-1 recovers molecular biomarkers of neuropathy and improves vascularisation in sciatic nerve of ob/ob mice. PLoS One. 2012;7:e32881. 22412941 10.1371/journal.pone.0032881 PMC3295786

[92] Zhou R Yazdi AS Menu P Tschopp J. A role for mitochondria in NLRP3 inflammasome activation. Nature. 2011;469:221–5. 21124315 10.1038/nature09663

[93] Xu F Qi H Li J. *Mycobacterium tuberculosis* infection up-regulates MFN2 expression to promote NLRP3 inflammasome formation. J Biol Chem. 2020;295:17684–97. 33454007 10.1074/jbc.RA120.014077 PMC7762945

[94] Park S Juliana C Hong S. The mitochondrial antiviral protein MAVS associates with NLRP3 and regulates its inflammasome activity. J Immunol. 2013;191:4358–66. 24048902 10.4049/jimmunol.1301170 PMC3848201

[95] Subramanian N Natarajan K Clatworthy MR Wang Z Germain RN. The adaptor MAVS promotes NLRP3 mitochondrial localization and inflammasome activation. Cell. 2013;153:348–61. 23582325 10.1016/j.cell.2013.02.054 PMC3632354

[96] Ichinohe T Yamazaki T Koshiba T Yanagi Y. Mitochondrial protein mitofusin 2 is required for NLRP3 inflammasome activation after RNA virus infection. Proc Natl Acad Sci USA. 2013;110:17963–8. 24127597 10.1073/pnas.1312571110 PMC3816452

[97] Sun Q Wang C Yan B. Jinmaitong ameliorates diabetic peripheral neuropathy through suppressing TXNIP/NLRP3 inflammasome activation in the streptozotocin-induced diabetic rat model. Diabetes Metab Syndr Obes. 2019;12:2145–55. 31802922 10.2147/DMSO.S223842 PMC6802560

[98] Jia M Wu C Gao F. Activation of NLRP3 inflammasome in peripheral nerve contributes to paclitaxel-induced neuropathic pain. Mol Pain. 2017;13:1744806917719804. 28714351 10.1177/1744806917719804 PMC5562344

[99] Zheng T Wang Q Bian F. Salidroside alleviates diabetic neuropathic pain through regulation of the AMPK-NLRP3 inflammasome axis. Toxicol Appl Pharmacol. 2021;416:115468. 33639149 10.1016/j.taap.2021.115468

[100] Liu Y-P Shao S-J Guo H-D. Schwann cells apoptosis is induced by high glucose in diabetic peripheral neuropathy. Life Sci. 2020;248:117459. 32092332 10.1016/j.lfs.2020.117459

[101] Zhang T Zhang D Zhang Z. Alpha-lipoic acid activates AMPK to protect against oxidative stress and apoptosis in rats with diabetic peripheral neuropathy. Hormones (Athens). 2023;22:95–105. 36289188 10.1007/s42000-022-00413-7

[102] Youle RJ Karbowski M. Mitochondrial fission in apoptosis. Nat Rev Mol Cell Biol. 2005;6:657–63. 16025099 10.1038/nrm1697

[103] Bossy-Wetzel E Barsoum MJ Godzik A Schwarzenbacher R Lipton SA. Mitochondrial fission in apoptosis, neurodegeneration and aging. Curr Opin Cell Biol. 2003;15:706–16. 14644195 10.1016/j.ceb.2003.10.015

[104] Jenner A Peña‐Blanco A Salvador-Gallego R. DRP1 interacts directly with BAX to induce its activation and apoptosis. EMBO J. 2022;41:e108587. 35023587 10.15252/embj.2021108587 PMC9016351

[105] Peña-Blanco A García-Sáez AJ. Bax, Bak and beyond – mitochondrial performance in apoptosis. FEBS J. 2018;285:416–31. 28755482 10.1111/febs.14186

[106] Peng L Men X Zhang W. Dynamin-related protein 1 is implicated in endoplasmic reticulum stress-induced pancreatic β-cell apoptosis. Int J Mol Med. 2011;28:161–9. 21537829 10.3892/ijmm.2011.684

[107] Wu Y-B Shi L-L Wu Y-J. Protective effect of gliclazide on diabetic peripheral neuropathy through Drp-1 mediated-oxidative stress and apoptosis. Neurosci Lett. 2012;523:45–9. 22732450 10.1016/j.neulet.2012.06.038

[108] Schmeichel AM Schmelzer JD Low PA. Oxidative injury and apoptosis of dorsal root ganglion neurons in chronic experimental diabetic neuropathy. Diabetes. 2003;52:165–71. 12502508 10.2337/diabetes.52.1.165

[109] Cheng Y-C Chu L-W Chen J-Y. Loganin attenuates high glucose-induced Schwann cells pyroptosis by inhibiting ROS generation and NLRP3 inflammasome activation. Cells. 2020;9:1948. 32842536 10.3390/cells9091948 PMC7564733

[110] Pang B Zhang L-L Li B Sun F-X Wang Z-D. BMP5 ameliorates diabetic peripheral neuropathy by augmenting mitochondrial function and inhibiting apoptosis in Schwann cells. Biochem Biophys Res Commun. 2023;643:69–76. 36587524 10.1016/j.bbrc.2022.12.071

[111] Suen D-F Norris KL Youle RJ. Mitochondrial dynamics and apoptosis. Genes Dev. 2008;22:1577–90. 18559474 10.1101/gad.1658508 PMC2732420

[112] Martorell-Riera A Segarra-Mondejar M Muñoz JP. Mfn2 downregulation in excitotoxicity causes mitochondrial dysfunction and delayed neuronal death. EMBO J. 2014;33:2388–407. 25147362 10.15252/embj.201488327 PMC4253527

[113] Lee Y-j Jeong S-Y Karbowski M Smith CL Youle RJ. Roles of the mammalian mitochondrial fission and fusion mediators Fis1, Drp1, and Opa1 in apoptosis. Mol Biol Cell. 2004;15:5001–11. 15356267 10.1091/mbc.E04-04-0294 PMC524759

[114] Cheng C Zochodne DW. Sensory neurons with activated caspase-3 survive long-term experimental diabetes. Diabetes. 2003;52:2363–71. 12941777 10.2337/diabetes.52.9.2363

[115] Wai T Langer T. Mitochondrial dynamics and metabolic regulation. Trends Endocrinol Metab. 2016;27:105–17. 26754340 10.1016/j.tem.2015.12.001

[116] Mizushima N Komatsu M. Autophagy: renovation of cells and tissues. Cell. 2011;147:728–41. 22078875 10.1016/j.cell.2011.10.026

[117] Youle RJ Narendra DP. Mechanisms of mitophagy. Nat Rev Mol Cell Biol. 2011;12:9–14. 21179058 10.1038/nrm3028 PMC4780047

[118] Stotland A Gottlieb RA. Mitochondrial quality control: easy come, easy go. Biochim Biophys Acta. 2015;1853:2802–11. 25596427 10.1016/j.bbamcr.2014.12.041 PMC4501896

[119] Geisler S Holmström KM Skujat D. PINK1/Parkin-mediated mitophagy is dependent on VDAC1 and p62/SQSTM1. Nat Cell Biol. 2010;12:119–31. 20098416 10.1038/ncb2012

[120] Shirihai OS Song M Dorn GW. How mitochondrial dynamism orchestrates mitophagy. Circ Res. 2015;116:1835–49. 25999423 10.1161/CIRCRESAHA.116.306374 PMC4443843

[121] Choi S-J Kim S Lee WS Kim DW Kim C-S Oh S-H. Autophagy dysfunction in a diabetic peripheral neuropathy model. Plast Reconstr Surg. 2023;151:355–64. 36355029 10.1097/PRS.0000000000009844

[122] Prasad M Jayaraman S Eladl MA. A comprehensive review on therapeutic perspectives of phytosterols in insulin resistance: a mechanistic approach. Molecules. 2022;27:1595. 35268696 10.3390/molecules27051595 PMC8911698

[123] Quirós PM Ramsay AJ Sala D. Loss of mitochondrial protease OMA1 alters processing of the GTPase OPA1 and causes obesity and defective thermogenesis in mice. EMBO J. 2012;31:2117–33. 22433842 10.1038/emboj.2012.70 PMC3343468

[124] Sebastián D Hernández-Alvarez MI Segalés J. Mitofusin 2 (Mfn2) links mitochondrial and endoplasmic reticulum function with insulin signaling and is essential for normal glucose homeostasis. Proc Natl Acad Sci USA. 2012;109:5523–8. 22427360 10.1073/pnas.1108220109 PMC3325712

[125] Kulkarni SS Joffraud M Boutant M. Mfn1 deficiency in the liver protects against diet-induced insulin resistance and enhances the hypoglycemic effect of metformin. Diabetes. 2016;65:3552–60. 27613809 10.2337/db15-1725

[126] Wang L Ishihara T Ibayashi Y. Disruption of mitochondrial fission in the liver protects mice from diet-induced obesity and metabolic deterioration. Diabetologia. 2015;58:2371–80. 26233250 10.1007/s00125-015-3704-7

[127] Bach D Naon D Pich S. Expression of Mfn2, the Charcot-Marie-Tooth neuropathy type 2A gene, in human skeletal muscle: effects of type 2 diabetes, obesity, weight loss, and the regulatory role of tumor necrosis factor α and interleukin-6. Diabetes. 2005;54:2685–93. 16123358 10.2337/diabetes.54.9.2685

[128] Hernandez Alvarez MI Thabet H Burns N. Subjects with early-onset type 2 diabetes show defective activation of the skeletal muscle PGC-1 {alpha}/mitofusin-2 regulatory pathway in response to physical activity. Diabetes Care. 2010;33:645–51. 20032281 10.2337/dc09-1305 PMC2827524

[129] Lo M-C Chen M-H Lee W-S. N ε-(carboxymethyl) lysine-induced mitochondrial fission and mitophagy cause decreased insulin secretion from β-cells. Am J Physiol Endocrinol Metab. 2015;309:E829–39. 26394662 10.1152/ajpendo.00151.2015

[130] Reinhardt F Schultz J Waterstradt R Baltrusch S. Drp1 guarding of the mitochondrial network is important for glucose-stimulated insulin secretion in pancreatic beta cells. Biochem Biophys Res Commun. 2016;474:646–51. 27154223 10.1016/j.bbrc.2016.04.142

[131] Cerqueira FM Chausse B Baranovski BM. Diluted serum from calorie-restricted animals promotes mitochondrial β-cell adaptations and protect against glucolipotoxicity. FEBS J. 2016;283:822–33. 26732506 10.1111/febs.13632

[132] Molina AJ Wikstrom JD Stiles L. Mitochondrial networking protects β-cells from nutrient-induced apoptosis. Diabetes. 2009;58:2303–15. 19581419 10.2337/db07-1781 PMC2750232

[133] Jheng H-F Tsai P-J Guo S-M. Mitochondrial fission contributes to mitochondrial dysfunction and insulin resistance in skeletal muscle. Mol Cell Biol. 2012;32:309–19. 22083962 10.1128/MCB.05603-11 PMC3255771

[134] Kluding PM Pasnoor M Singh R. The effect of exercise on neuropathic symptoms, nerve function, and cutaneous innervation in people with diabetic peripheral neuropathy. J Diabetes Complications. 2012;26:424–9. 22717465 10.1016/j.jdiacomp.2012.05.007 PMC3436981

[135] Balducci S Iacobellis G Parisi L. Exercise training can modify the natural history of diabetic peripheral neuropathy. J Diabetes Complications. 2006;20:216–23. 16798472 10.1016/j.jdiacomp.2005.07.005

[136] Dixit S Maiya AG Shastry B. Effect of aerobic exercise on peripheral nerve functions of population with diabetic peripheral neuropathy in type 2 diabetes: a single blind, parallel group randomized controlled trial. J Diabetes Complications. 2014;28:332–9. 24507164 10.1016/j.jdiacomp.2013.12.006

[137] Fealy CE Mulya A Lai N Kirwan JP. Exercise training decreases activation of the mitochondrial fission protein dynamin-related protein-1 in insulin-resistant human skeletal muscle. J Appl Physiol (1985). 2014;117:239–45. 24947026 10.1152/japplphysiol.01064.2013 PMC4122691

[138] Kang SWS Haydar G Taniane C. AMPK activation prevents and reverses drug-induced mitochondrial and hepatocyte injury by promoting mitochondrial fusion and function. PLoS One. 2016;11:e0165638. 27792760 10.1371/journal.pone.0165638 PMC5085033

[139] Shackelford DB Shaw RJ. The LKB1–AMPK pathway: metabolism and growth control in tumour suppression. Nat Rev Cancer. 2009;9:563–75. 19629071 10.1038/nrc2676 PMC2756045

[140] Hardie DG Ross FA Hawley SA. AMPK: a nutrient and energy sensor that maintains energy homeostasis. Nat Rev Mol Cell Biol. 2012;13:251–62. 22436748 10.1038/nrm3311 PMC5726489

[141] Herzig S Shaw RJ. AMPK: guardian of metabolism and mitochondrial homeostasis. Nat Rev Mol Cell Biol. 2018;19:121–35. 28974774 10.1038/nrm.2017.95 PMC5780224

[142] Ronnett GV Ramamurthy S Kleman AM Landree LE Aja S. AMPK in the brain: its roles in energy balance and neuroprotection. J Neurochem. 2009;109:17–23. 19393004 10.1111/j.1471-4159.2009.05916.x PMC2925428

[143] Shin SM Cho IJ Kim SG. Resveratrol protects mitochondria against oxidative stress through AMP-activated protein kinase-mediated glycogen synthase kinase-3β inhibition downstream of poly (ADP-ribose) polymerase-LKB1 pathway. Mol Pharmacol. 2009;76:884–95. 19620254 10.1124/mol.109.058479

[144] Melemedjian OK Asiedu MN Tillu DV. Targeting adenosine monophosphate-activated protein kinase (AMPK) in preclinical models reveals a potential mechanism for the treatment of neuropathic pain. Mol Pain. 2011;7:1744–70. 10.1186/1744-8069-7-70 PMC3186752 21936900

[145] Trewin AJ Berry BJ Wojtovich AP. Exercise and mitochondrial dynamics: keeping in shape with ROS and AMPK. Antioxidants (Basel, Switzerland). 2018;7:7. 29316654 10.3390/antiox7010007 PMC5789317

[146] Rovira-Llopis S Apostolova N Banuls C Muntané J Rocha M Victor VM. Mitochondria, the NLRP3 inflammasome, and sirtuins in type 2 diabetes: new therapeutic targets. Antioxid Redox Signal. 2018;29:749–91. 29256638 10.1089/ars.2017.7313

[147] Chowdhury SKR Dobrowsky RT Fernyhough P. Nutrient excess and altered mitochondrial proteome and function contribute to neurodegeneration in diabetes. Mitochondrion. 2011;11:845–54. 21742060 10.1016/j.mito.2011.06.007 PMC3375692

[148] Calcutt NA Smith DR Frizzi K. Selective antagonism of muscarinic receptors is neuroprotective in peripheral neuropathy. J Clin Invest. 2017;127:608–22. 28094765 10.1172/JCI88321 PMC5272197

[149] Roy Chowdhury SK Smith DR Saleh A. Impaired adenosine monophosphate-activated protein kinase signalling in dorsal root ganglia neurons is linked to mitochondrial dysfunction and peripheral neuropathy in diabetes. Brain. 2012;135:1751–66. 22561641 10.1093/brain/aws097 PMC3359752

[150] Toyama EQ Herzig S Courchet J. AMP-activated protein kinase mediates mitochondrial fission in response to energy stress. Science. 2016;351:275–81. 26816379 10.1126/science.aab4138 PMC4852862

[151] Jäger S Handschin C St.-Pierre J Spiegelman BM. AMP-activated protein kinase (AMPK) action in skeletal muscle via direct phosphorylation of PGC-1α. Proc Natl Acad Sci USA. 2007;104:12017–22. 17609368 10.1073/pnas.0705070104 PMC1924552

[152] Chandhok G Lazarou M Neumann B. Structure, function, and regulation of mitofusin-2 in health and disease. Biol Rev. 2018;93:933–49. 29068134 10.1111/brv.12378 PMC6446723

[153] Choi J Chandrasekaran K Inoue T Muragundla A Russell JW. PGC-1α regulation of mitochondrial degeneration in experimental diabetic neuropathy. Neurobiol Dis. 2014;64:118–30. 24423644 10.1016/j.nbd.2014.01.001 PMC4023837

[154] O’Mealey GB Berry WL Plafker SM. Sulforaphane is a Nrf2-independent inhibitor of mitochondrial fission. Redox Biol. 2017;11:103–10. 27889639 10.1016/j.redox.2016.11.007 PMC5126150

[155] Rambold AS Pearce EL. Mitochondrial dynamics at the interface of immune cell metabolism and function. Trends Immunol. 2018;39:6–18. 28923365 10.1016/j.it.2017.08.006

[156] Webb M Sideris DP Biddle M. Modulation of mitochondrial dysfunction for treatment of disease. Bioorg Med Chem Lett. 2019;29:1270–7. 30954429 10.1016/j.bmcl.2019.03.041

[157] Zhang Y Wang Y Xu J. Melatonin attenuates myocardial ischemia‐reperfusion injury via improving mitochondrial fusion/mitophagy and activating the AMPK-OPA1 signaling pathways. J Pineal Res. 2019;66:e12542. 30516280 10.1111/jpi.12542

[158] López-Lluch G. Mitochondrial activity and dynamics changes regarding metabolism in ageing and obesity. Mech Ageing Dev. 2017;162:108–21. 27993601 10.1016/j.mad.2016.12.005

[159] Morrison RS Kinoshita Y Johnson MD Guo W Garden GA. p53-dependent cell death signaling in neurons. Neurochem Res. 2003;28:15–27. 12587660 10.1023/a:1021687810103

[160] Wang W Cheng X Lu J. Mitofusin-2 is a novel direct target of p53. Biochem Biophys Res Commun. 2010;400:587–92. 20804729 10.1016/j.bbrc.2010.08.108

[161] Zhou H Du W Li Y. Effects of melatonin on fatty liver disease: The role of NR 4A1/DNA-PK cs/p53 pathway, mitochondrial fission, and mitophagy. J Pineal Res. 2018;64:e12450. 10.1111/jpi.12450 28981157

[162] Shi X Pi L Zhou S. Activation of sirtuin 1 attenuates high glucose-induced neuronal apoptosis by deacetylating p53. Front Endocrinol. 2018;9:274. 10.3389/fendo.2018.00274 PMC5985296 29892266

[163] Ben Y Hao J Zhang Z. Astragaloside IV inhibits mitochondrial-dependent apoptosis of the dorsal root ganglion in diabetic peripheral neuropathy rats through modulation of the SIRT1/p53 signaling pathway. Diabetes Metab Syndr Obes. 2021;14:1647–61. 33883914 10.2147/DMSO.S301068 PMC8055373

[164] Sugimoto K Nishizawa Y Horiuchi S Yagihashi S. Localization in human diabetic peripheral nerve of Nɛ-carboxymethyllysine-protein adducts, an advanced glycation endproduct. Diabetologia. 1997;40:1380–7. 9447944 10.1007/s001250050839

[165] Lukic IK Humpert PM Nawroth PP Bierhaus A. The RAGE pathway: activation and perpetuation in the pathogenesis of diabetic neuropathy. Ann N Y Acad Sci. 2008;1126:76–80. 18448798 10.1196/annals.1433.059

[166] Wang X Li Q Han X Gong M Yu Z Xu B. Electroacupuncture alleviates diabetic peripheral neuropathy by regulating glycolipid-related GLO/AGEs/RAGE axis. Front Endocrinol. 2021;12:655591. 10.3389/fendo.2021.655591 PMC8290521 34295304

[167] Zhou K Chen J Wu J. Atractylenolide III ameliorates cerebral ischemic injury and neuroinflammation associated with inhibiting JAK2/STAT3/Drp1-dependent mitochondrial fission in microglia. Phytomedicine. 2019;59:152922. 30981186 10.1016/j.phymed.2019.152922

[168] Egaña-Gorroño L López-Díez R Yepuri G. Receptor for advanced glycation end products (RAGE) and mechanisms and therapeutic opportunities in diabetes and cardiovascular disease: insights from human subjects and animal models. Front Cardiovasc Med. 2020;7:37. 10.3389/fcvm.2020.00037 PMC7076074 32211423

[169] Wada R Yagihashi S. Role of advanced glycation end products and their receptors in development of diabetic neuropathy. Ann N Y Acad Sci. 2005;1043:598–604. 16037282 10.1196/annals.1338.067

[170] Chen H Chan DC. Mitochondrial dynamics – fusion, fission, movement, and mitophagy – in neurodegenerative diseases. Hum Mol Genet. 2009;18:R169–76. 19808793 10.1093/hmg/ddp326 PMC2758711

[171] Ziegler D Nowak H Kempler P Vargha P Low PA. Treatment of symptomatic diabetic polyneuropathy with the antioxidant α-lipoic acid: a meta-analysis. Diabet Med. 2004;21:114–21. 14984445 10.1111/j.1464-5491.2004.01109.x

[172] Su B Wang X Zheng L Perry G Smith MA Zhu X. Abnormal mitochondrial dynamics and neurodegenerative diseases. Biochim Biophys Acta. 2010;1802:135–42. 19799998 10.1016/j.bbadis.2009.09.013 PMC2790543

[173] Dai C-Q Guo Y Chu X-Y. Neuropathic pain: the dysfunction of Drp1, mitochondria, and ROS homeostasis. Neurotox Res. 2020;38:553–63. 32696439 10.1007/s12640-020-00257-2

[174] Ferrari LF Chum A Bogen O Reichling DB Levine JD. Role of Drp1, a key mitochondrial fission protein, in neuropathic pain. J Neurosci. 2011;31:11404–10. 21813700 10.1523/JNEUROSCI.2223-11.2011 PMC3157245

[175] Chen L Huang J Li X-C. High-glucose induced mitochondrial dynamics disorder of spinal cord neurons in diabetic rats and its effect on mitochondrial spatial distribution. Spine. 2019;44:E715–22. 30601355 10.1097/BRS.0000000000002952

[176] Wang Q Zhang M Torres G. Metformin suppresses diabetes-accelerated atherosclerosis via the inhibition of Drp1-mediated mitochondrial fission. Diabetes. 2017;66:193–205. 27737949 10.2337/db16-0915 PMC5204316

[177] Kim B Kim J-S Yoon Y Santiago MC Brown MD Park J-Y. Inhibition of Drp1-dependent mitochondrial division impairs myogenic differentiation. Am J Physiol Regul Integr Comp Physiol. 2013;305:R927–38. 23904108 10.1152/ajpregu.00502.2012

[178] Smith G Gallo G. To mdivi-1 or not to mdivi-1: is that the question? Dev Neurobiol. 2017;77:1260–8. 28842943 10.1002/dneu.22519 PMC5654677

[179] Zhang Q Song W Zhao B. Quercetin attenuates diabetic peripheral neuropathy by correcting mitochondrial abnormality via activation of AMPK/PGC-1α pathway in vivo and in vitro. Front Neurosci. 2021;15:636172. 33746703 10.3389/fnins.2021.636172 PMC7966726

[180] Ding M Feng N Tang D. Melatonin prevents Drp1-mediated mitochondrial fission in diabetic hearts through SIRT 1-PGC 1α pathway. J Pineal Res. 2018;65:e12491. 29575122 10.1111/jpi.12491 PMC6099285

[181] Zhu J Yang X Li X Han S Zhu Y Xu L. Tang Luo Ning, a traditional Chinese compound prescription, ameliorates schwannopathy of diabetic peripheral neuropathy rats by regulating mitochondrial dynamics in vivo and in vitro. Front Pharmacol. 2021;12:650448. 34054529 10.3389/fphar.2021.650448 PMC8160508

